# Supplementary material for: Taming the chaos gently: a predictive alignment learning rule in recurrent neural networks
Source: Nat Commun. 2025 Jul 23;16:6784. doi: 10.1038/s41467-025-61309-9 (PMC12287340; doi:10.1038/s41467-025-61309-9)
Supplement: Supplementary file 1 — Supplementary Information [file 41467_2025_61309_MOESM1_ESM.pdf]

Supplementary Information for:

Taming the chaos gently: a Predictive Alignment learning rule in recurrent neural networks

Toshitake Asabuki[1,2,3] & Claudia Clopath[1]

[1] Department of Bioengineering, Imperial College London, London, UK.

[2] RIKEN Center for Brain Science, RIKEN ECL Research Unit

[3] RIKEN Cluster for Pioneering Research, Japan

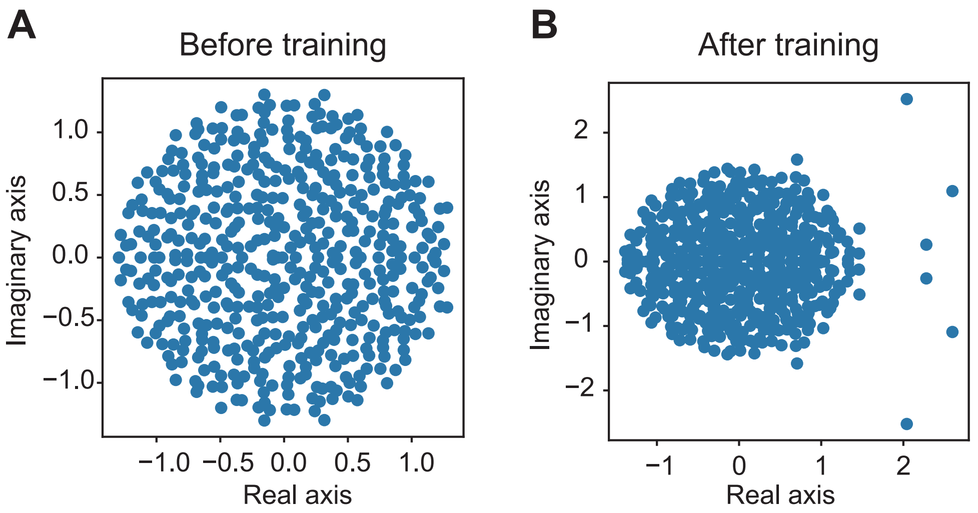

Supplementary Figure 1. Eigenvalue spectrum of the recurrent connections. (A) The eigenvalues are uniformly distributed within a circle, since the initial recurrent connections were generated by Gaussian distribution. (B) The spectrum of weights after training shows that most eigenvalues still lie on a circle in the complex plane, while only a few eigenvalues lie outside the circle.

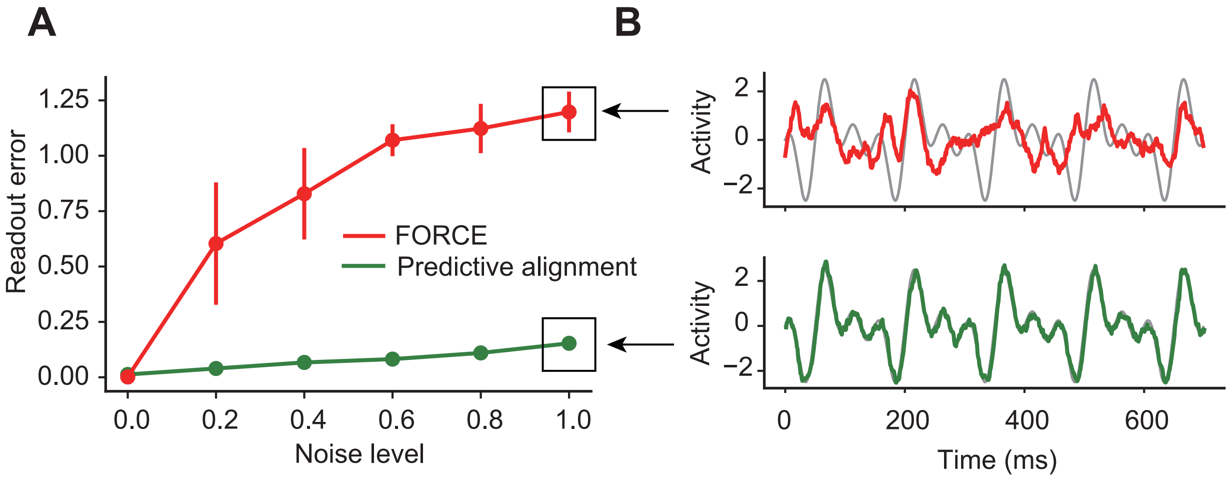

Supplementary Figure 2. Robustness of predictive alignment against noise. (A) Networks with different strengths of noise were trained with the FORCE (red) and the predictive alignment (green) with the patterned target signal. Error bars stand for s.d.s over 20 independent simulations. (B) Example readout activities during the late phase of training are shown. Colors are the same as in A. The gray traces represent the target signal.

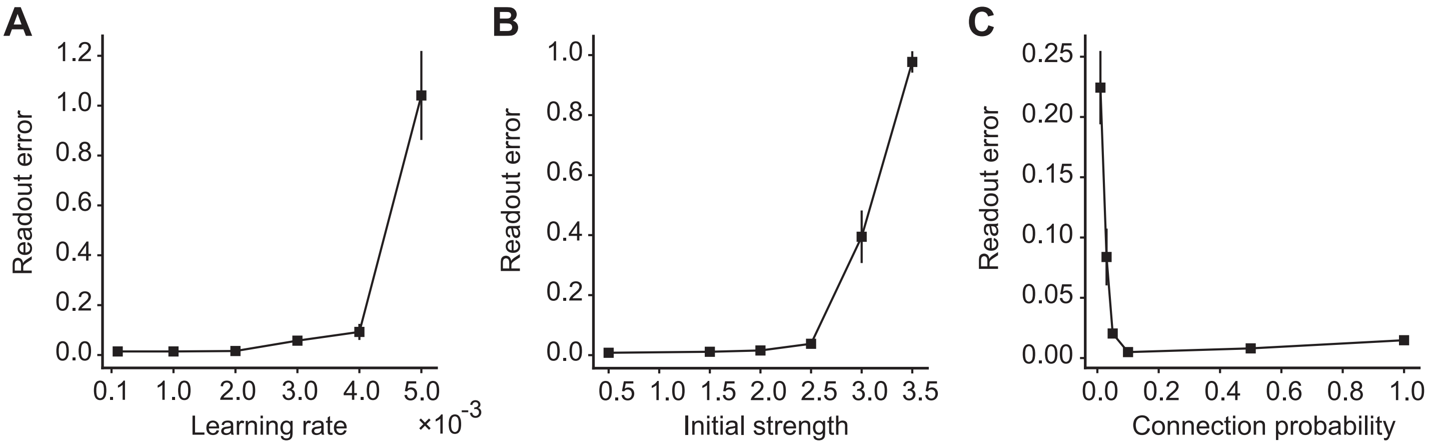

Supplementary Figure 3. Robustness to hyperparameter choices. (A) The networks were trained with different levels of learning rates. (B) Same as A, but trained with different degrees of the initial strength of the plastic recurrent weights. (C) Same as A, but trained with different degrees of connection probabilities of the plastic recurrent weights. Error bars represent SEs across five independent simulations.

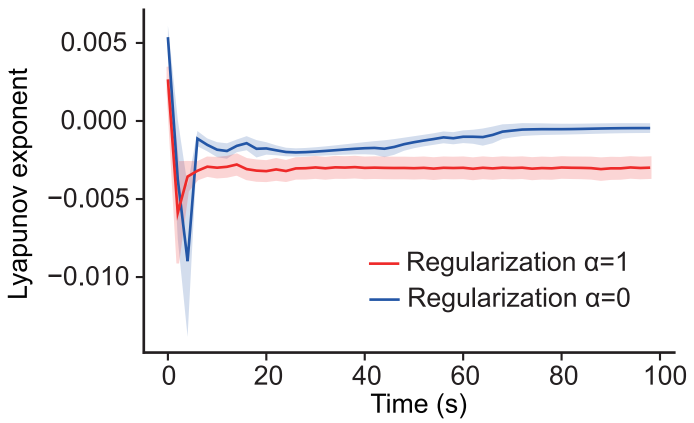

Supplementary Figure 4. Dynamics of the Lyapunov exponent during learning. Stronger alignment induced by regularization leads to more effective suppression of chaos.

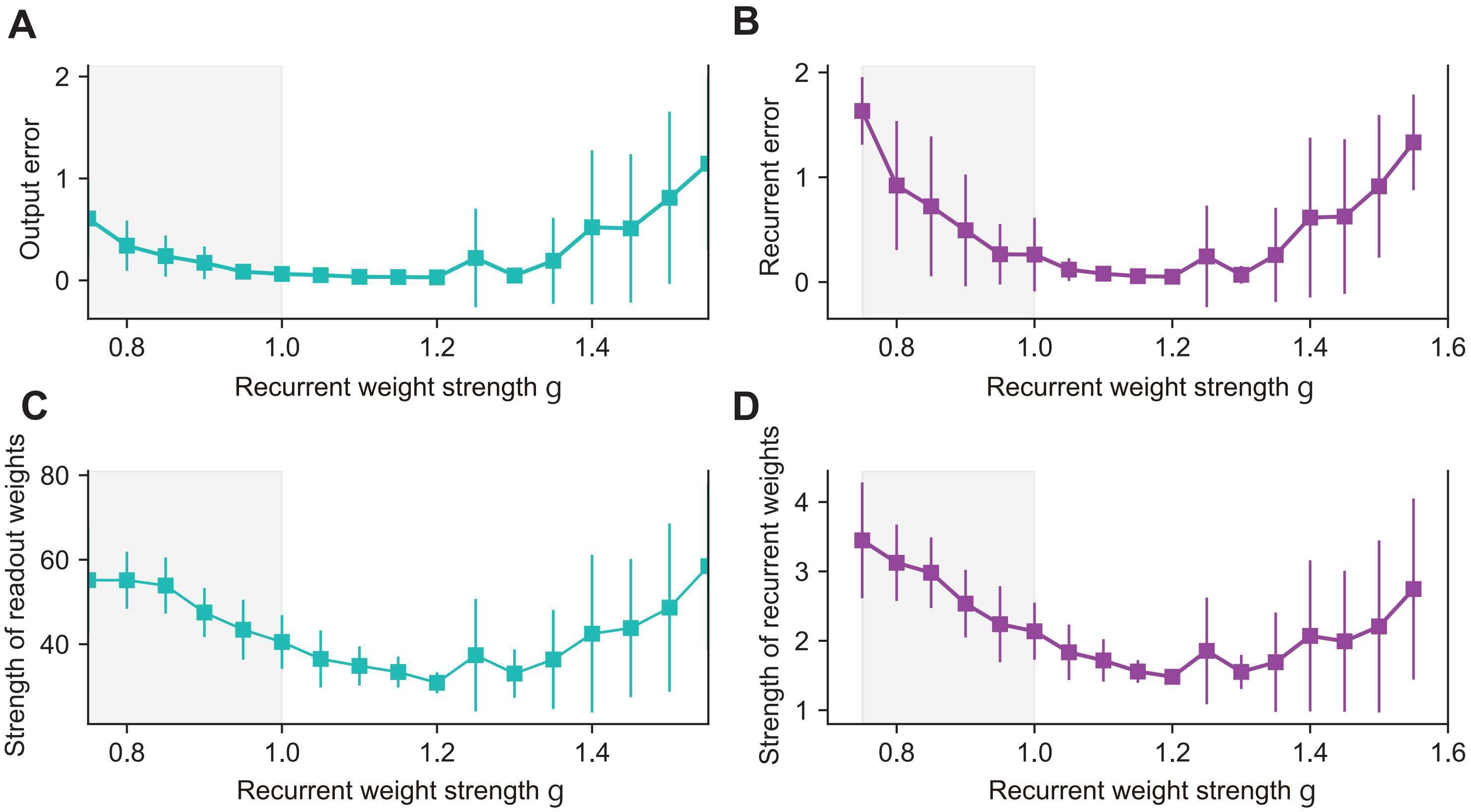

Supplementary Figure 5. Crucial role of edge of chaos. (A) Output error of the trained network over various varue of recurrent strength. (B) Same as in A, but for the recurrent error. (C) Strength of trained readout weights are shown over various varue of recurrent strength. (D) Same as in C, but for the recurrent connection. Error bars stand for s.d.s over 20 independent simulations. In all figures, g=1 indicates that the network is on the edge of chaos.

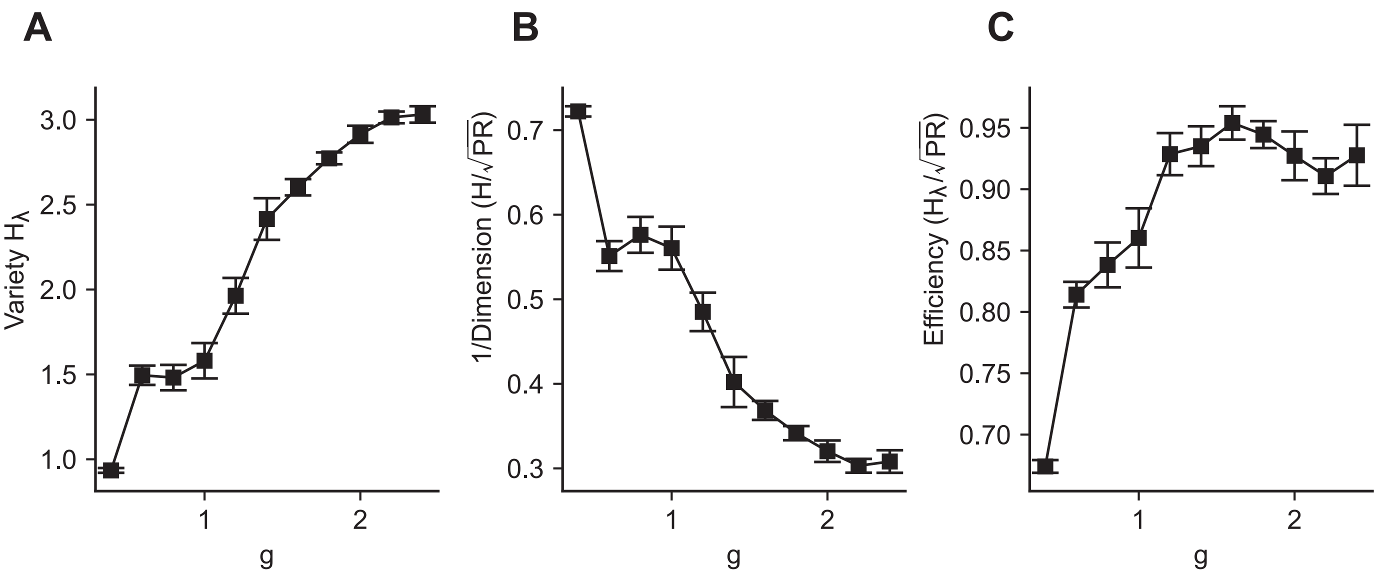

Supplementary Figure 6. Advantages of the edge of chaos. (A) Neural activity diversity increases as recurrent connection strength g increases. (B) The inverse of the dimensionality of population activity decreases with increasing recurrent connection strength. (C) Computational efficiency is maximized when the network operates at the edge of chaos.

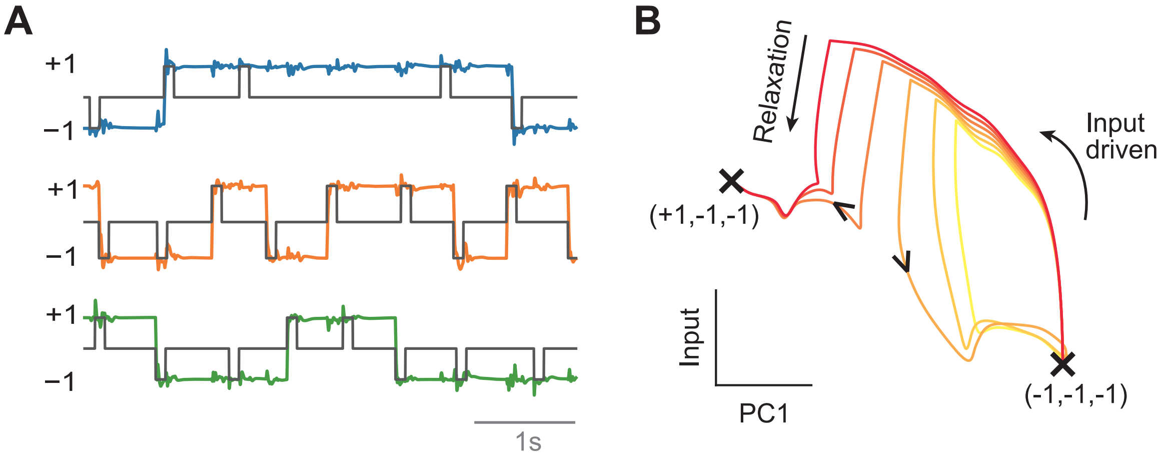

Supplementary Figure 7. Learning fixed point attractors. (A) Example three inputs (black) and outputs (blue, orange, and green) are shown. (B) Low dimensional network dynamics are shown. Network dynamics perturbed with the varying strength of inputs shows that a saddle point mediates the transitions between attractors.

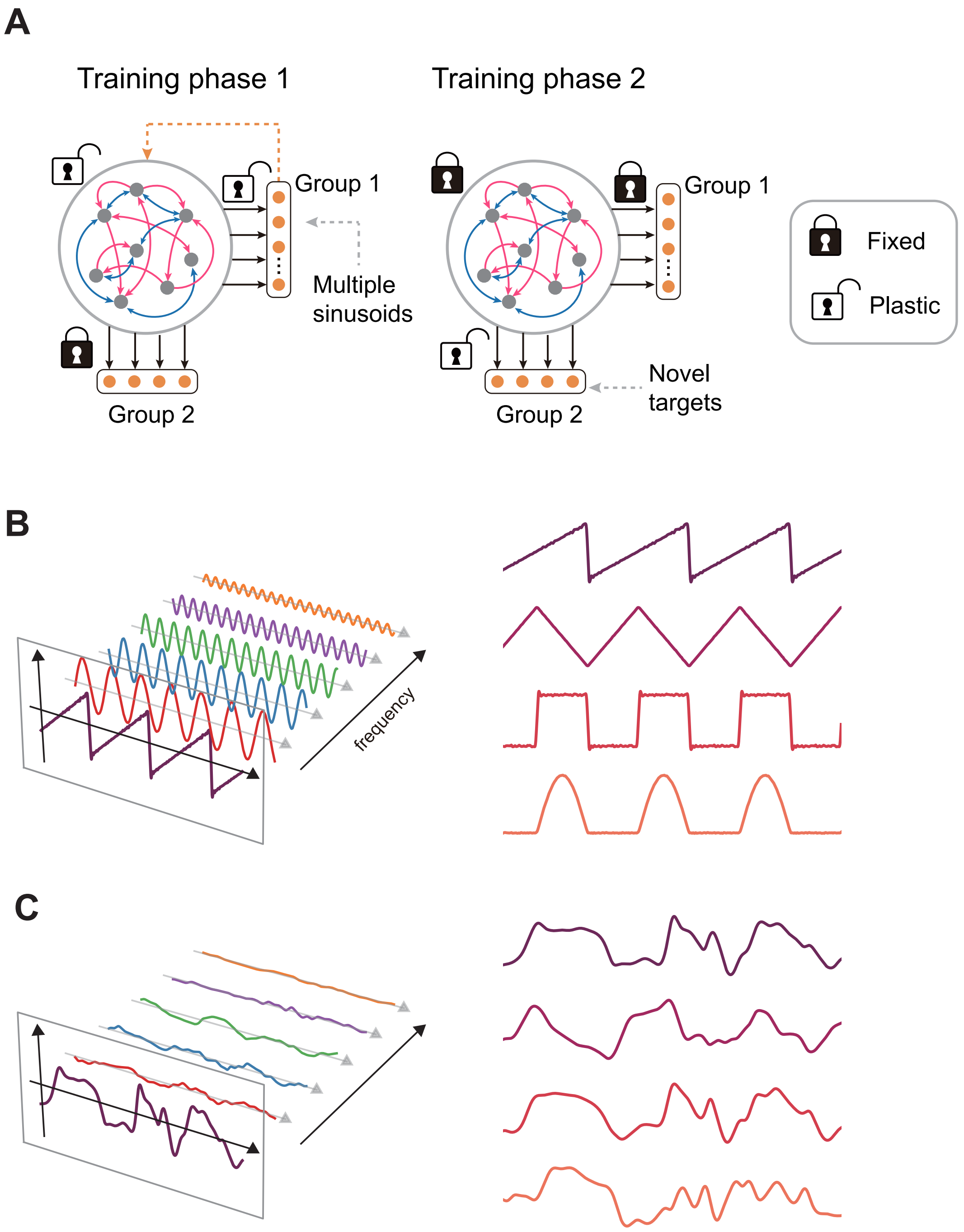

Supplementary Figure 8. Generalization of learned representations. (A) The readout units were divided into two groups, and the training phase consisted of two stages. During the first stage of learning, readouts in the first group were trained to generate sinusoids with multiple frequencies (colored sinusoids in B). All readout weights projecting to the first group of readouts and the recurrent connections were trained in the first stage. In the second stage of learning, only the readout weights projecting to the second goup of readouts were trained to generate complex signals (discontinuous signals in B). (B)The model generalized multiple sinusoids and various complex signals simultaneously. (C) Same as in B, but without initial training.

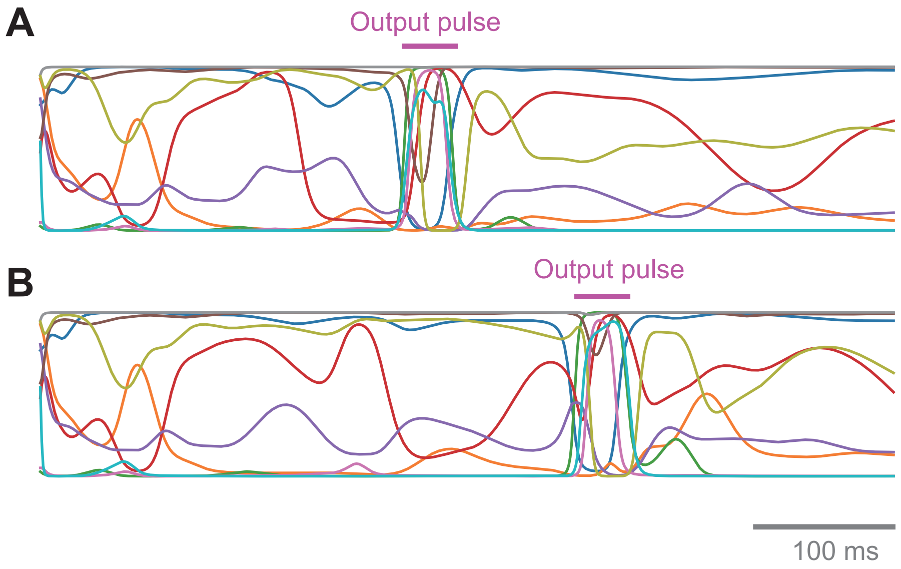

Supplementary Figure 9. Neural activities for performing Ready-Set-Go task. (A) Example activities of 10 recurrent neurons during the task with the time delay of 100 ms are shown. Neurons showed phasic responses during the output generated a pulse. (B) Same as A, but the time delay of 160 ms.

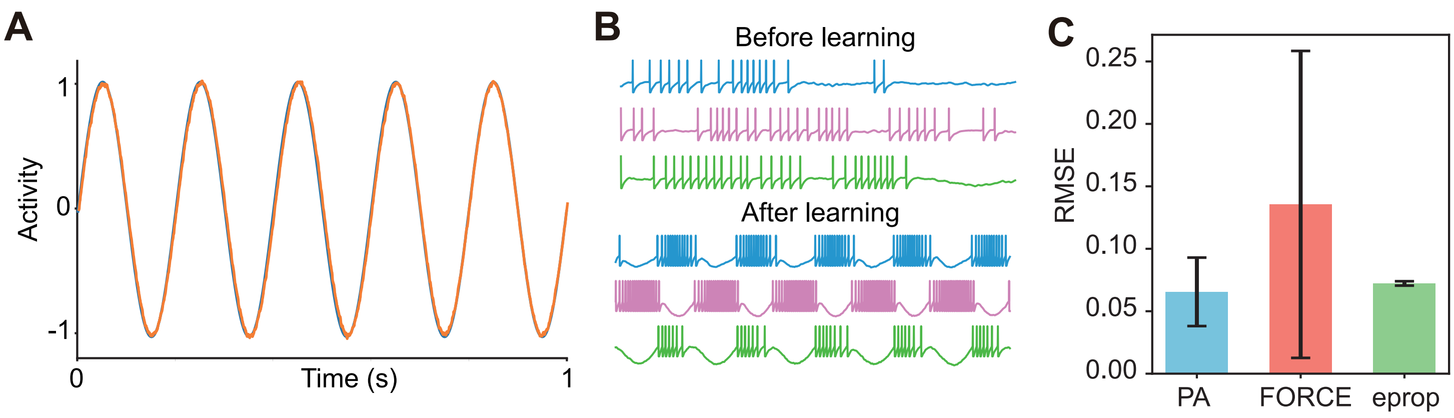

Supplementary Figure 10. The spiking recurrent network was trained to learn periodic target signal. (A) The blue trace represents the target signal, and the orange line represents the output. (B) Example activities of spiking network neurons before (top) and after training (bottom) with the Predictive Alignment are shown. The modified recurrent connections generate the patterned network activity. (C) Performance comparison. Error bars indicate the standard deviations (s.d.s) across 10 independent simulations. Note that we did not extensively tune the hyperparameters for either e-prop or FORCE. Furtheremore, the performance of FORCE was unstable since we used 500 neurons across all models—a number that may not be sufficient to fully realize FORCE's potential.

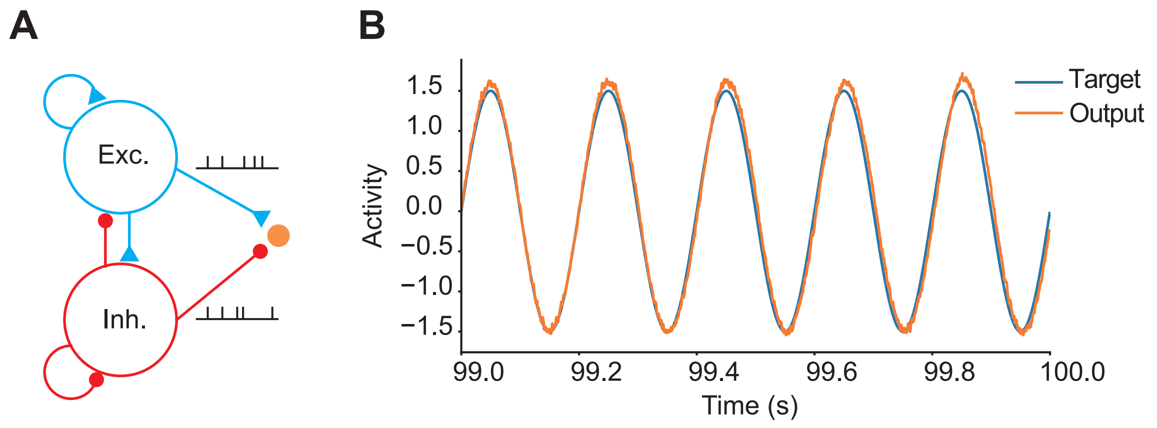

Supplementary Figure 11. Network model consisting of distinct excitatory and inhibitory populations. (A) Schematic of the model. (B) Output after learning.
