# Supplementary material for: Taming the chaos gently: a predictive alignment learning rule in recurrent neural networks
Source: Nat Commun. 2025 Jul 23;16:6784. doi: 10.1038/s41467-025-61309-9 (PMC12287340; doi:10.1038/s41467-025-61309-9)
Supplement: Supplementary file 4 — Description: Target movie and RNN-generated replay [file 41467_2025_61309_MOESM4_ESM.pdf]

Title: Supplementary Movie 1

Description: Target movie and RNN-generated replay
